# Smoking reduces surfactant protein D and phospholipids in patients with and without chronic obstructive pulmonary disease

**DOI:** 10.1186/1471-2466-10-53

**Published:** 2010-10-25

**Authors:** Jayaji M Moré, Dennis R Voelker, Lori J Silveira, Michael G Edwards, Edward D Chan, Russell P Bowler

**Affiliations:** 1 Department of Medicine, National Jewish Health, Denver, CO, USA; 2 Department of Physics, Stanford University, Stanford, CA, USA; 3 Department of Medicine, University of Colorado at Denver, Denver, CO, USA

## Abstract

**Background:**

Pulmonary surfactant D (SP-D) has important regulatory functions for innate immunity and has been implicated as a biomarker for chronic obstructive pulmonary disease (COPD). We hypothesized that COPD patients would have reduced bronchoalveolar lavage (BAL) fluid SP-D levels compared to healthy smoking and non-smoking controls.

**Methods:**

BAL SP-D and phospholipids were quantified and corrected for dilution in 110 subjects (65 healthy never smokers, 23 smokers with normal spirometry, and 22 smokers with COPD).

**Results:**

BAL SP-D was highest in never smokers (mean 51.9 μg/mL ± 7.1 μg/mL standard error) compared to both smokers with normal spirometry (16.0 μg/mL ± 11.8 μg/mL) and subjects with COPD (19.1 μg/mL ± 12.9 μg/mL; P < 0.0001). Among smokers with COPD, BAL SP-D correlated significantly with FEV_1_% predicted (R = 0.43; P < 0.05); however, the strongest predictor of BAL SP-D was smoking status. BAL SP-D levels were lowest in current smokers (12.8 μg/mL ± 11.0 μg/mL), intermediate in former smokers (25.2 μg/mL ± 14.2 μg/mL; P < 0.008), and highest in never smokers. BAL phospholipids were also lowest in current smokers (6.5 nmol ± 1.5 nmol), intermediate in former smokers (13.1 nmol ± 2.1 nmol), and highest in never smokers (14.8 nmol ± 1.1 nmol; P < 0.0001).

**Conclusions:**

These data suggest that smokers, and especially current smokers, exhibit significantly reduced BAL SP-D and phospholipids compared to nonsmokers. Our findings may help better explain the mechanism that leads to the rapid progression of disease and increased incidence of infection in smokers.

## Background

Chronic obstructive pulmonary disease (COPD) is the fourth leading cause of death in the United States [1], and worldwide, both COPD morbidity and mortality are expected to increase dramatically in the next ten years. Cigarette smoke is the major risk factor for COPD; however, not all smokers are diagnosed with COPD. One possible explanation for why some smokers do not develop clinically important COPD is the role that host factors play in limiting damage from smoking. One of the limitations in COPD research has been a lack of lung-specific biomarkers, particularly ones that could play a role in both pathogenesis and therapy. Surfactant protein D (SP-D) is a strong candidate for such a biomarker [2].

SP-D is a large multimeric, calcium binding glycoprotein that is a member of the collectin family. The protein serves as an innate immune regulatory molecule and is produced predominantly in the lungs. SP-D promotes the elimination of pathogens by its ability to recognize carbohydrate structures on the surface of large numbers of bacteria, viruses and fungi. Both environmental and genetic factors have been shown to contribute to SP-D expression [3].

Currently there are conflicting data regarding the role of SP-D in the pathogenesis of COPD. Some polymorphisms in the SP-D gene have been associated with COPD [4,5]; however, replication studies have not confirmed these findings [6]. Serum levels of SP-D have been reported to be higher in COPD patients [7], and those with high serum SP-D have more COPD exacerbations [8], but other reports have found that SP-D is not affected by smoking status [9]. The use of SP-D as a biomarker for COPD has also been suggested in a report that found regular inhalation of salmeterol and fluticasone lowers serum SP-D levels in COPD patients [10].

Although SP-D is predominantly a lung protein, there are very few studies that have reported lung SP-D in smokers. Hirama et al. reported that smoking increases SP-D lung mRNA expression and protein in bronchoalveolar lavage (BAL) in a mouse smoking model [11]. In contrast, human studies have reported that smokers have lower BAL SP-D compared to non-smokers [12,13]. These data suggest that SP-D could be a good biomarker for COPD and that smoking may alter SP-D levels in the lung; however, most of these studies were limited by small sample size, lack of inclusion of a group of former smokers, no description of lung function, lack of correction for dilution during the BAL procedure, and no measurement of total phospholipids. In this study, we overcame size limitations by evaluating a larger study group of 110 subjects, including former smokers. Additionally, we performed spirometry and corrected BAL SP-D by measuring dilution of plasma to BAL urea as well as total phospholipids. The goals of this study were to examine SP-D and phospholipid levels in normal nonsmokers as well as current and former smokers with and without COPD to determine whether significant changes are associated with smoke exposure and COPD.

## Methods

### Subjects

All subjects were studied under protocols approved by the Institutional Review Board at National Jewish Health (HS-1725) with guidelines recommended by the National Institutes of Health. Written informed consent was obtained for all subjects. Subjects were free of significant chronic disease (other than COPD) that might put them at risk during bronchoscopy (e.g. known congestive heart failure, MI in past year, history of pneumonia in the past 6 months, or bronchitis/COPD exacerbation within the past month) and recruited from the community by word of mouth and written advertisements. Subjects were excluded if they had a history of lung cancer within or were suspected of having lung cancer after their bronchoscopy. A total of 110 subjects were recruited in Denver, Colorado. Smokers were defined as having smoked at least 10 pack years. Never smokers were defined as having smoked less than 100 cigarettes over their lifetime. Smokers were self-defined as being current smokers or former smokers with a quit date of at least one month prior to bronchoscopy. Current smokers had their last cigarette a mean of 2.1 hours prior to bronchoscopy (range: 1 - 3 hours). Post bronchodilator spirometry and the diagnosis of COPD were made using Global initiative for chronic Obstructive Lung Disease (GOLD) criteria [14]. The percent predicted FEV_1 _represents the maximum volume of air expired one second after the onset of full expiration compared to that predicted for one's age, sex, height, and race. Among the COPD group there were 6 categorized as GOLD 1, 8 categorized GOLD 2, 4 categorized as GOLD 3, and 4 categorized as GOLD 4. Comorbidities of more than 3% in the population included: diabetes (N = 5); hypercholesterolemia (N = 8); hypertension (N = 12); and a remote history of pneumonia (N = 9). 101 subjects self-reported as non-Hispanic (92 White, 8 black, and 1 more than one race), 5 subjects self reported as Hispanic (5 White now classified as American Indian), and 3 had unreported race and ethnicity. Active inhaled corticosteroid use was 6/22 in COPD subjects, 1/23 smokers without COPD, and none of the non-smoking controls. None of the non-smoking controls and only 7/22 COPD subjects and 2/23 smoking controls reported a history of exacerbations within the past year.

### BAL and Blood Collection

Six mL of blood was withdrawn from the antecubital vein into a sterile 13 × 100 mm sodium heparin Vacutainer Plus (BD, New Jersey). The sample was immediately centrifuged at 2100 × g for 10 minutes at room temperature and then cell free aliquots were frozen at -80°C. BAL fluid was obtained from a 60 ml saline lavage with the bronchoscope wedged in the anterior segment of the right upper lobe. Samples were centrifuged at 1500 × g for 10 minutes at 4°C to remove cells and cellular debris and the supernatant was immediately frozen at -80°C. All reagents were supplied by Sigma Chemical (St. Louis, MO) unless otherwise noted.

### Determination of SP-D in BAL

Human SP-D was measured by sandwich enzyme-linked immunosorbent assay (ELISA) using polyclonal human SP-D antibodies developed in rabbit. SP-D was detected using anti-human SP-D IgG and a horseradish peroxidase (HRP) conjugated secondary antibody. Standards contained recombinant human SP-D protein produced in Chinese hamster ovary (CHO) cells as previously described [15]. Standards ranged from 40 ng/mL to 0.625 ng/mL. BAL samples were diluted serially in ratios ranging from 1:5 to 1:40 in buffer containing 3% non-fat dry milk in phosphate buffered saline (PBS) with 1% Trition X-100. The samples were quantified by light absorbance at 405 nm approximately 40 minutes after addition of developer. The developer used was the Invitrogen (Carlsbad, CA) ABTS Chromogen/Substrate Solution for ELISA. Each sample was measured in triplicate and the values averaged. The coefficient of variation (CFV%) of absorbance among triplicate samples was noted (mean: 3.65% ± 2.79%; range: 0.095% to 13.63%); a sample was retested if the CFV% (absorbance) of its BAL SP-D measurement exceeded 13.63%.

### Determination of Phospholipids

Lipids were extracted from a 1 mL sample of BAL by the method of Bligh and Dyer [16]. Lipid phosphorus was determined by the method of Rouser [17]. The samples were quantified by light absorbance at 820 nm with a standard curve ranging from 1 to 35 nmol sodium phosphate.

### Determination of Blood Urea

Blood urea nitrogen (BUN) was measured using the Stanbio Laboratory (Boerne, TX) Urea Nitrogen (BUN) Liqui-UV Procedure No. 2020 Kit. The blood samples were diluted two-fold with 5 μL of ultra pure distilled water. In addition to the diluted sample, 200 μL of BUN reagent was added to a 96-well microtiter plate. The samples were quantified by light absorbance at 340 nm, 5 minutes after the BUN reagent was added. Urea standards ranged from 0.469 to 30 mg/dL. Each assay was preformed in triplicate. Final urea concentrations were recorded as the average of the three triplicate samples.

### Statistical Analysis

SP-D levels were natural log transformed to better approximate model assumptions in all cases. SAS version 9.2 and JMP version 8.0 for Macintosh (SAS Institute, Cary, NC) was used to compute means and standard deviations. In the primary analysis, SP-D levels were corrected for BAL dilution by multiplying measured values by each sample's dilution factor (BUN/BAL urea nitrogen). The mean dilution factor was 85.0 ± 72.1 (range: 11.5 to 499.5), and there were no differences in dilution factor among groups. In secondary analysis, BAL SP-D was expressed per nanomole of lipid phosphate. Means were compared by ANOVA. A significant P value was considered to be <0.05. Linear regression was used for continuous variables (including: sex, age, pack years, FEV_1 _and FVC).

## Results

Demographics including age, sex, pack years, and lung function sorted by smoking history of the 110 subjects participating in this study are listed in Table 1. Additional demographic data included: BMI (mean 26, standard deviation 3.6) and years smoked (mean 37 years, standard deviation 10.6 years). The distribution of men and women was not significantly different among groups, but the never smokers were younger than both the smokers with and without COPD (P < 0.001). Of the smokers, those with COPD were also significantly older than those without COPD (P < 0.001). Smokers with COPD were more likely to be former smokers compared to smokers without COPD (P < 0.05) and also had higher reported pack years (P < 0.01). By definition, the smokers with COPD had worse lung function compared to the never smoker and smoker control group. Because of the potential effects of current smoking on SP-D and phospholipid levels, we also analyzed the smoker groups based on whether they were current or former smokers. Current smokers were younger (56 ± 8 years) compared to former smokers (63 ± 8 years; P < 0.05). Current smokers also had slightly better lung function compared to former smokers (FEV_1_% predicted 83% ± 21% versus 64% ± 30%; P < 0.05). However, there were no significant differences in pack years smoking between current (43 ± 22 pack years) and former smokers (56 ± 29 pack years).

**Table 1:** Demographics of study participants

	Never Smoker (N = 65)	Smokers (N = 45)	
		**Control (N = 23)**	**COPD (N = 22)**
Age	28 ± 8	53 ± 5	64 ± 9
Sex (m/f)	36/29	18/5	14/8
Current Smokers	0%	78%	41%
Pack Years	0	36 ± 14	60 ± 29
FEV_1 _post bronchodilator (l)	4.2 ± 0.9	3.2 ± 0.7	1.7 ± 0.7
FEV_1 _(% predicted)	103% ± 11%	95% ± 13%	56% ± 21%
FEV_1_/FVC	0.85 ± 0.06	0.79 ± 0.05	0.53 ± 0.12

Shown are means ± standard deviations; P values in text

**Figure 1:**
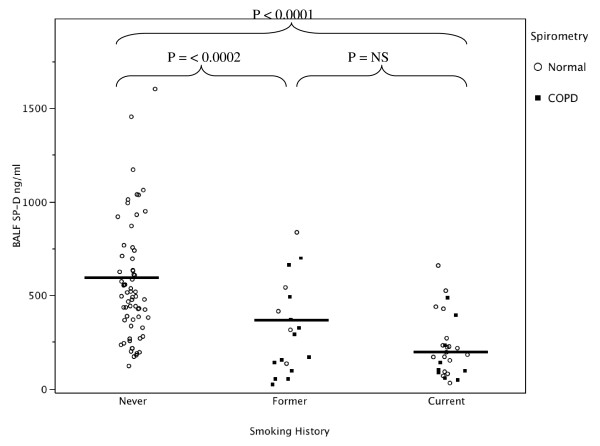
**Bronchoalveolar lavage fluid SP-D levels in never, former, and current smokers with normal lung function or COPD as defined by spirometery**. The never smokers had significantly higher SP-D levels in their lavage fluid compared to former and current smokers.

**Figure 2:**
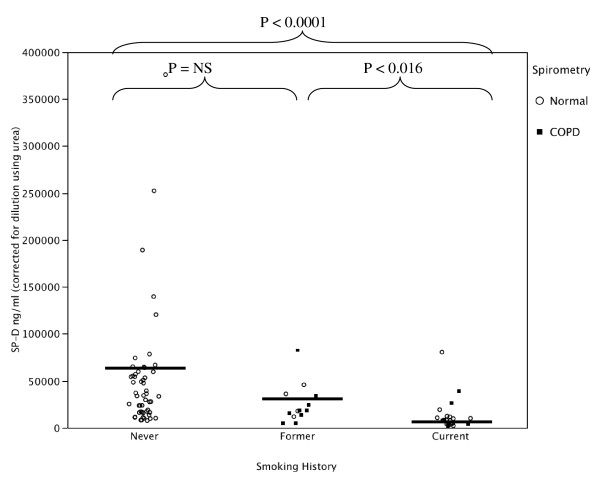
**Bronchoalveolar lavage fluid SP-D levels corrected for dilution in never, former, and current smokers with normal lung function or COPD as defined by spirometry**. The dilution factor of the BAL was calculated by the ratio of plasma/BAL urea. The never smokers had significantly higher SP-D levels in their lavage fluid compared to former and current smokers.

**Figure 3:**
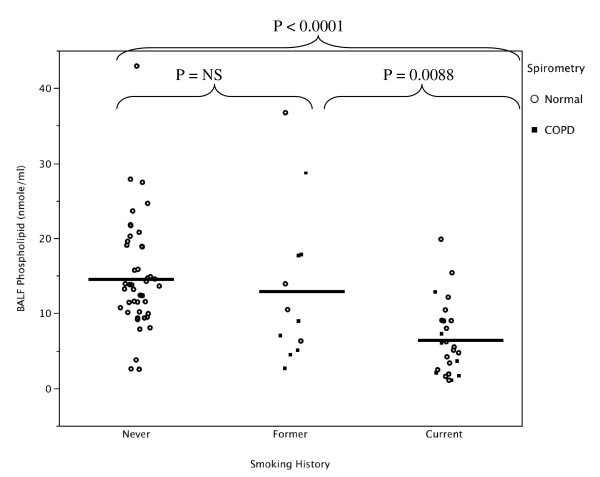
**Bronchoalveolar lavage fluid phospholipid levels in never, former, and current smokers with normal lung function or COPD as defined by spirometry**. Lipids were extracted and phospholipids measured. The never smokers had significantly more phospholipids in their lavage fluid compared to former and current smokers. Current smokers had significantly lower levels compared to former smokers.

**Figure 4:**
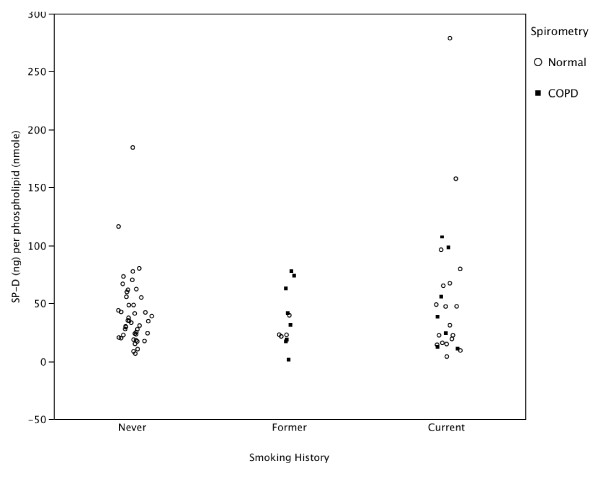
**Bronchoalveolar lavage fluid SP-D corrected for total phospholipid in never, former, and current smokers with normal lung function or COPD as defined by spirometry**. There were no significant differences among groups.

Compared to smokers with or without COPD, the never smoker group had significantly higher recovered (natural log transformed) BAL SP-D with (Figure 1 and Additional File 1: Supplemental Figure S1) and without (Figure 2 and Additional File 1: Supplemental Figure S2) correction for dilution by urea (Table 2 and Additional File 1: Supplemental Table S1). BAL recovered phospholipids were also significantly higher in the never smokers compared to the smoker groups (Figure 3 and Additional File 1: Supplemental Figure S3). When SP-D was corrected for phospholipids, the difference between the never smokers and the smokers was not significant (Figure 4 and Additional File 1: Supplemental Figure S4). Although the non-smokers were younger than smokers, there were no significant correlations between age and SP-D in the non-smokers. There were no statistically significant associations between surfactant or phospholipids and other demographic variables such as comorbidities, BMI, years smoked, race or ethnicity, active inhaled corticosteroid use, or a history of exacerbations.

**Table 2:** Bronchoalveolar lavage fluid SP-D and phospholipids

	Never Smoker	Smokers		
		**Control**	**COPD**	**P**
BAL SP-D (uncorrected) ng/ml	558 ± 309	287 ± 204	235 ± 203	<0.0001
BAL SP-D (corrected) μg/ml	51.9 ± 64.3	16.0 ± 19.3	19.1 ± 20.5	<0.0001
BAL phospholipid (nmol/ml)	14.8 ± 7.4	8.9 ± 7.8	8.5 ± 7.7	<0.0001
BAL SP-D ng/phospholipid nmol	42.2 ± 31.5	52.1 ± 61.8	44.8 ± 33.3	N.S.

Shown are means ± standard deviations; P values were calculated using ln transformed data (see Additional File 1: Supplemental Table S1).

There were no statistically significant differences in SP-D (corrected or uncorrected) or phospholipid levels between the smokers with COPD and the smokers without COPD (Table 2 and Additional File 1: Supplemental Table S1); however, there was a correlation between FEV_1_% predicted and recovered BAL SP-D (R = 0.43; P < 0.05) among COPD subjects. Compared to former smokers, current smokers had significantly less BAL phospholipids (6.5 ± 1.4 versus 13.3 ± 2.0 nmol/ml; P = 0.0088; Additional File 1: Supplemental Figure S3). Current smokers also had lower SP-D compared to the former smokers (13 ± 17 versus 25 ± 21 μg/ml corrected for dilution; P = 0.016). In a multiple regression model that included age, FEV_1_% predicted, and smoking history, smoking status (current or former) was a significant predictor for BAL SP-D (P < 0.05) and phospholipids (P < 0.001).

## Discussion

The main finding of this study was that current smoking was associated with lower levels of BAL SP-D and phospholipids compared to former and never smokers. Although we did not find a statistical difference in the BAL SP-D or phospholipids levels between COPD patients and smokers without COPD, lower BAL SP-D was associated with worse lung function in COPD subjects. A likely explanation for why smokers without COPD did not have significantly higher SP-D and phospholipids compared to COPD subjects is that there were significantly more current smokers in the control group compared to the COPD group. The multiple regression analysis suggested that current smoking was the strongest predictor of diminished BAL SP-D and phospholipid levels.

The first study of the effect of smoking on phospholipids was reported by Finley and Ladman [18], who noted lower BAL return of phospholipids in smokers. Interestingly they noted that three of the smokers who quit had a rapid rise in BAL phospholipids after as little as one month. The earliest study to document the effects of smoking on human BAL SP-D was a small study of 8 smokers and 12 nonsmokers by Honda et al [13]. These investigators reported similar results to us in that current smokers had lower BAL SP-D and phospholipids compared to never smokers. Unlike our study, the investigators did not study former smokers nor did they report on lung function or age of subjects; however, similar to us, they did find that correcting surfactant protein levels for phospholipid content made differences in BAL surfactant proteins A and D non-significant. Our findings are also consistent with Betsuyaku et al [12] who reported BAL SP-D in 22 nonsmokers and 82 smokers. The subjects in this study were almost exclusively males and investigators did not correct for dilution nor did they report total phospholipid content. They also found that SP-D did not change with age alone, however, it was decreased in middle- aged or elderly smokers when compared with similarly aged nonsmokers.

Although we did not measure serum SP-D, several investigators have found a different relationship between serum surfactant proteins in smokers and COPD patients. In a study of 237 healthy smoking and non-smoking subjects, serum levels of SP-A were significantly higher in male smokers compared to male nonsmokers [19]. There was a non-significant trend for lower SP-A values in young nonsmoking males and females compared to older subjects. Mutti et al [7] found that active smokers and COPD subjects had higher SP-D serum levels compared to control never smokers; however, there was no correlation with GOLD stage nor were there reports of the effect of age on SP-D levels. The reason for the opposite relationship between BAL and blood SP-D is unclear. Sin et al has proposed that the lower levels of BAL SP-D may be due to increased transmigration of SP-D from the alveolar space into blood [2]. There are, however, few data to support this theory. Genetic studies of twins have suggested that serum SP-D levels are influenced by a combination of environmental effects (e^2 ^= 0.19), additive genetic effects (h^2 ^= 0.42), and the effect of a single nucleotide polymorphism (Met11Thr) (q^2 ^= 0.39) [3]. None of the BAL studies have reported genotyping of the SP-D gene; however, a large replication genetic study did not confirm a role for this polymorphism in COPD [6].

Animal studies have shed some light on the role of SP-D in smoking and the effects of aging, but may be difficult to extrapolate to humans. Hirama et al [11] found that mice exposed to cigarette smoke for six months had increased lung SP-D mRNA expression and increased BAL SP-D protein in the lungs. Furthermore over-expression of SP-D in A549 cells was found to increase survival in vitro after exposure to 4% cigarette smoke extract. Reanalysis of SP-D gene expression from normal lung (NCBI GEO database accession #GSE1643 [20]) revealed no differences in SP-D gene expression among healthy never smokers, current smokers or non-smokers; however, there was a significant negative correlation to age (Spearman's rho = -0.60995, P = 0.003). Thus animal models, which showed increased SP-D gene expression after smoke exposure, and human gene expression studies, which show no difference in gene expression by smoking status, contrast with human SP-D protein quantification studies that have consistently found decreased BAL SP-D protein in smokers. The reasons for the differences between the mouse model and human gene expression studies are unclear, but suggest that lower levels of protein in the BAL of human smokers might be the result of increased clearance or increased degradation.

SP-D is recognized as an important regulator of innate immunity capable of binding pathogens and facilitating phagocytosis [21]. The protein also exerts direct microbicidal activity on selected bacteria and fungi [22,23]. Lower levels of SP-D caused by cigarette smoking may thus weaken lung immunity. Mice harboring SP-D null alleles show reduced bacterial clearance and elevated basal levels of inflammation [24,25]. *In vitro *SP-D can suppress inflammatory responses elicited by LPS and peptidogylcan [26,27]. Thus, chronically reduced levels of SP-D are expected to cause defects in microbial recognition and impaired suppression of inflammation. Cigarette smoking as has other strong effects on innate immunity of the lung independent of surfactants and phospholipids [28]. These effects include impairment of T-cell function leading to susceptibility of infection, dysfunctional ciliary epithelium, and reduction of macrophage and neutrophils phagocytic capabilities.

Recent evidence also implicates anionic pulmonary surfactant phospholipids as important negative regulators of TLR4 activation and inflammation as well as respiratory syncytial virus induced inflammation [29,30]. Thus, reduction of both SP-D and surfactant phospholipids in smokers is likely to increase inflammation and predispose these individuals to certain viral infections.

There are several limitations of this and previous studies. Foremost is that the mechanisms by which smoking is associated with lower recovered BAL SP-D and phospholipids is unknown. Schmekel et al have postulated that alveolar macrophages in smokers may increase clearance of phospholipids [31] or alternatively there could be more leakage of surfactant proteins and lipids into blood [2,18]. Another possible explanation for our observed differences in BAL phospholipids is the younger age of controls; however, the correlation between BAL phospholipids and age has only been demonstrated in children < 8 years of age [32], which is consistent with our inability to demonstrate a relationship between aging and phospholipids or SP-D levels in our adult never smokers. Nevertheless, both an age and a smoking-related decrease in SP-D and phospholipids might explain why COPD is predominantly a disease of elderly smokers. Furthermore, because current smokers were allowed their last cigarette two hours prior to the collection of BAL, the unknown acute effects of smoking on SP-D and phospholipids may have impacted our results. Finally, in this study, the relationship between COPD and BAL SP-D was probably understated because there were significantly more current smokers in the smokers with normal lung function group, compared to smokers with COPD.

## Conclusion

In this study we demonstrate that smoking, especially current smoking, and worse lung function are associated with low BAL SP-D and phospholipid levels. Reduced phospholipids and surfactant proteins may predispose smokers to infections or defective suppression of inflammation in response to pathogen exposure, ambient LPS levels, or LPS that can be found in cigarette smoke. This may lead to a more rapid decline in lung function than is normally associated with aging.

## Abbreviations

COPD: chronic obstructive pulmonary disease; BAL: bronchoalveolar lavage; SP-D: surfactant protein-D; GOLD: global initiative for chronic obstructive lung disease; BUN: blood urea nitrogen

## Competing interests

The authors declare that they have no competing interests.

## Authors' contributions

RB was responsible for funding, design, analysis, and discussion. He had full access to all of the data in the study and takes responsibility for the integrity of the data and the accuracy of the data analysis. JM performed all experiments and was responsible for writing the manuscript and critical revision for important intellectual content. DV provided technical assistance with the surfactant and phospholipids assays, participated in manuscript writing, and provided critical revision for important intellectual content. LS conducted the statistical analysis. ME contributed to writing and reviewing the manuscript. EC contributed BAL samples and participated in writing and reviewing the manuscript.

All authors have read and approved the final manuscript.

## Pre-publication history

The pre-publication history for this paper can be accessed here:

http://www.biomedcentral.com/1471-2466/10/53/prepub

## Supplementary Material

Additional file 1 **Natural log transformed data used for analysis**. A table summarizing natural log transformed data and four figures showing the distribution of natural log transformed data by group Click here for file
