# Supplementary material for: Smoking reduces surfactant protein D and phospholipids in patients with and without chronic obstructive pulmonary disease
Source: BMC Pulm Med. 2010 Oct 25;10:53. doi: 10.1186/1471-2466-10-53 (PMC2987951; doi:10.1186/1471-2466-10-53)

Additional File 1

Smoking reduces surfactant protein D and phospholipids in patients with and without COPD

Jayaji M More^1,2^, Dennis Voelker^1^, Lori J Silveira^1^, Michael Edwards^3^, Edward D Chan^1^, Russell P **Bowler^1^**

^1^Dept of Medicine, National Jewish Health, Denver, CO, USA. ^3^Dept of Medicine, University of Colorado at Denver, Denver, CO, USA, ^2^Stanford University, Stanford, CA, USA.

CORRESPONDENCE:

R. P. Bowler, Division of Pulmonary Medicine, Dept of Medicine, National Jewish Health, 1400 Jackson Street, Room K715a, Denver, CO 80206, USA. Fax: 1 3032702249. E-mail: [BowlerR@njhealth.org](mailto:BowlerR@njhealth.org)

Supplement Table S1

|  | Never Smoker | Smokers | |  |
| --- | --- | --- | --- | --- |
|  |  | Control | COPD | P |
| ln (BAL SP-D (uncorrected) ng/ml) | 6.18 ± .56 | 5.40 ± .95 | 5.07 ± .95 | < 0.0001 |
| ln (BAL SP-D (corrected) µg/ml)) | 10.4 ± .90 | 9.2 ± .94 | 9.36 ± 1.09 | < 0.0001 |
| ln (BAL phospholipid (nmole/ml)) | 2.57 ± .55 | 1.87 ± .86 | 1.75 ± .94 | < 0.0001 |
| ln(BAL SP-D ng)/ln(phospholipid nmole) | 3.53 ± .67 | 3.50 ± .96 | 3.40 ± 1.14 | N.S. |

Shown are natural log transformed (ln) means ± standard deviations;

Supplement Figure S1: Bronchoalveolar lavage fluid SP-D levels (natural log transformed) in never, former, and current smokers with normal lung function or COPD as defined by spirometry. The never smokers had significantly higher SP-D levels in their lavage fluid compared to former and current smokers.

Supplement Figure S2: Bronchoalveolar lavage fluid SP-D levels (natural log transformed) corrected for dilution in never, former, and current smokers with normal lung function or COPD as defined by spirometry. The dilution factor of the BAL was calculated by the ratio of plasma/BAL urea. The never smokers had significantly higher SP-D levels in their lavage fluid compared to former and current smokers.

Supplement Figure S3: Bronchoalveolar lavage fluid phospholipid levels (natural log transformed) in never, former, and current smokers with normal lung function or COPD as defined by spirometry. Lipids were extracted and phospholipid measured. The never smokers had significantly more phospholipids in their lavage fluid compared to former and current smokers. Current smokers had significantly lower levels compared to former smokers

Supplement Figure S4: Bronchoalveolar lavage fluid SP-D (natural log transformed) corrected for total phospholipid (natural log transformed) in never, former, and current smokers with normal lung function or COPD as defined by spirometry. There were no significant differences among groups.


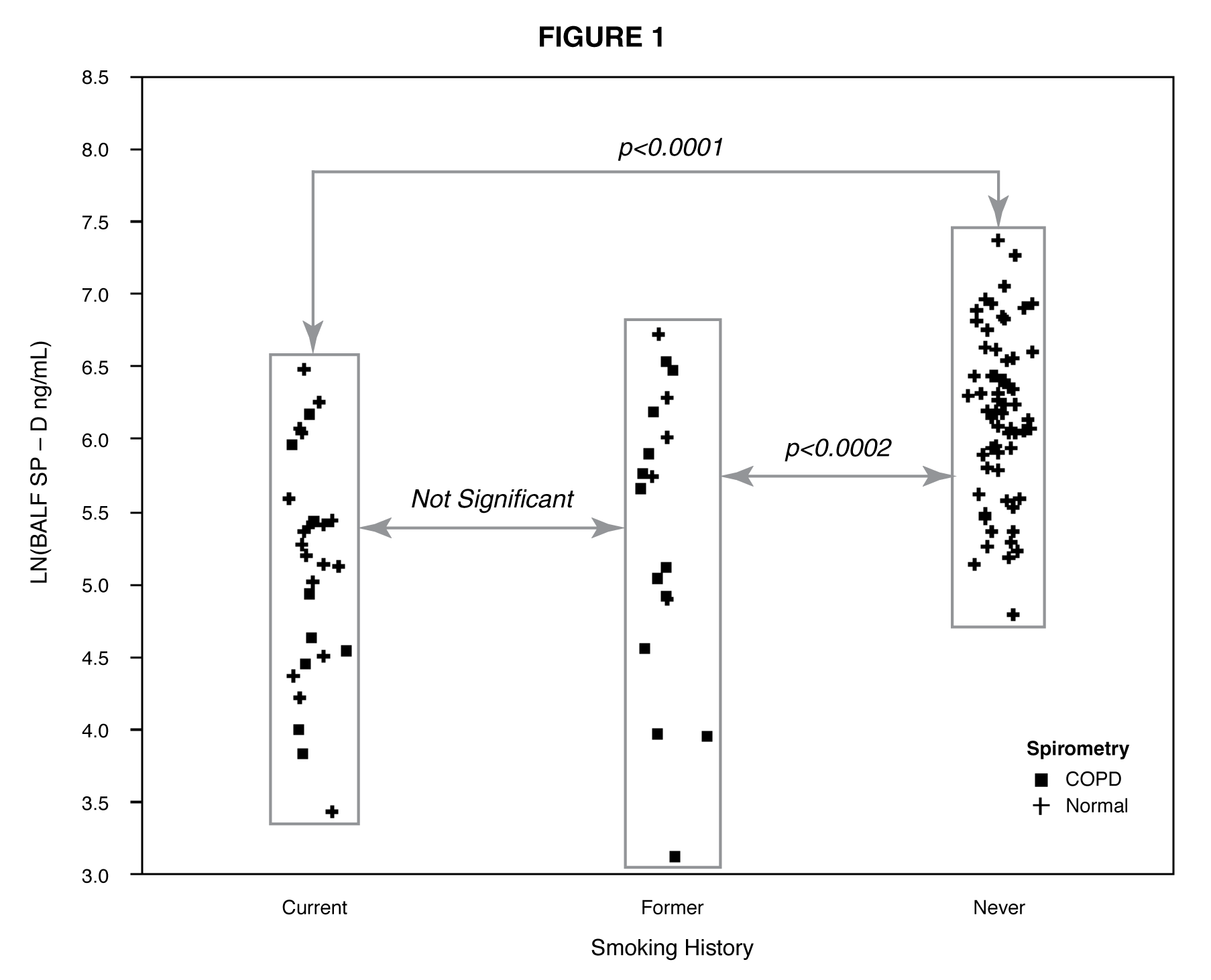


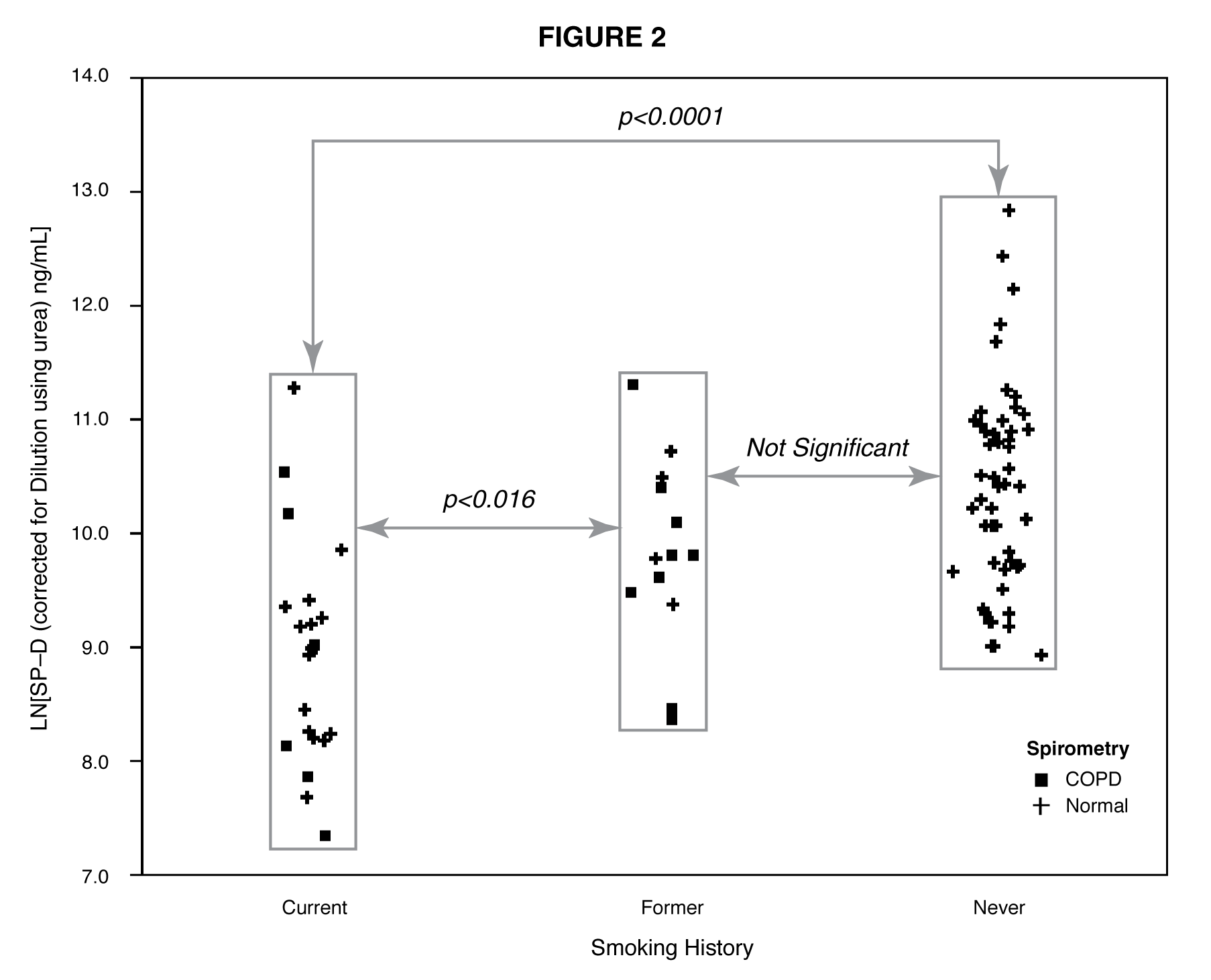


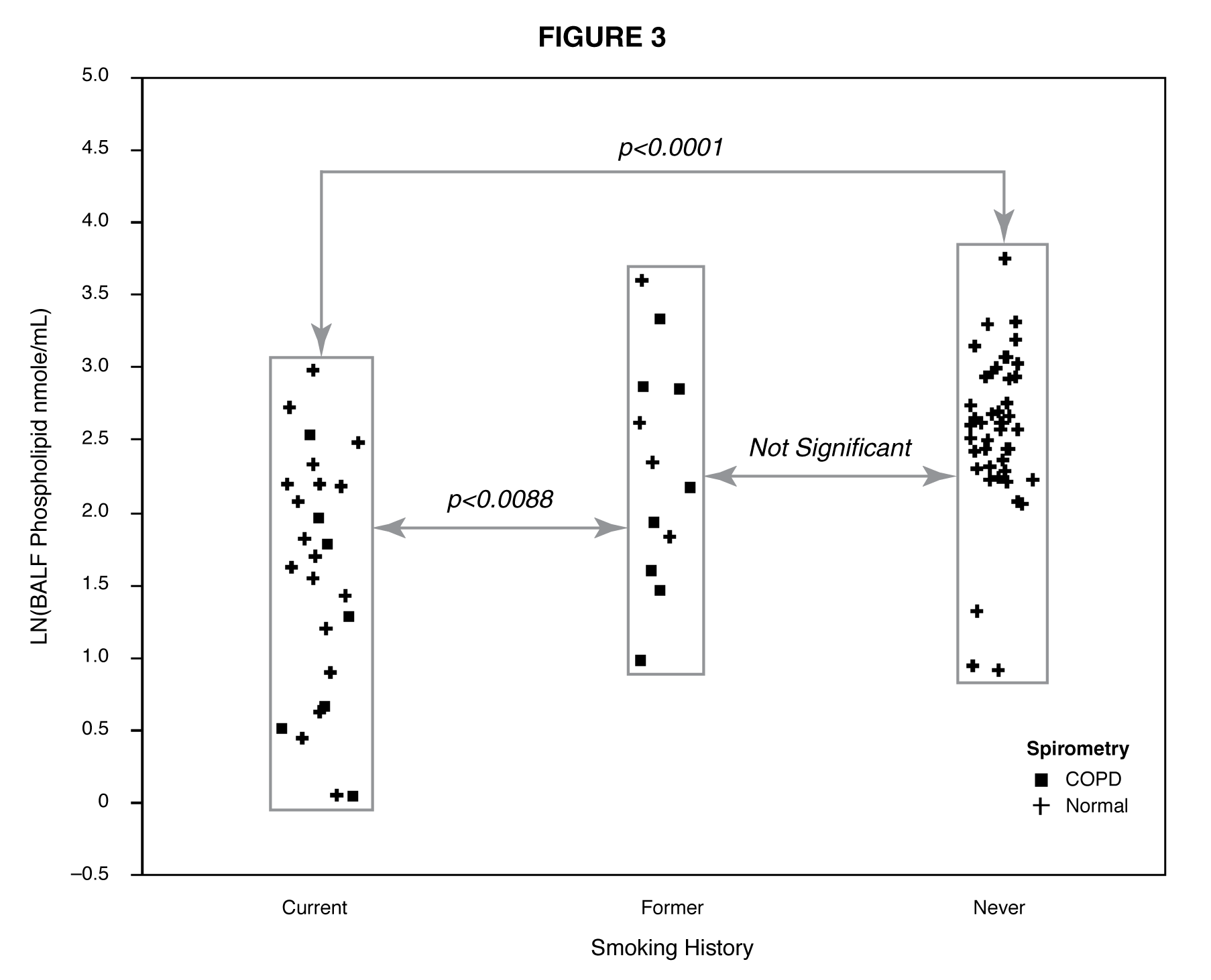


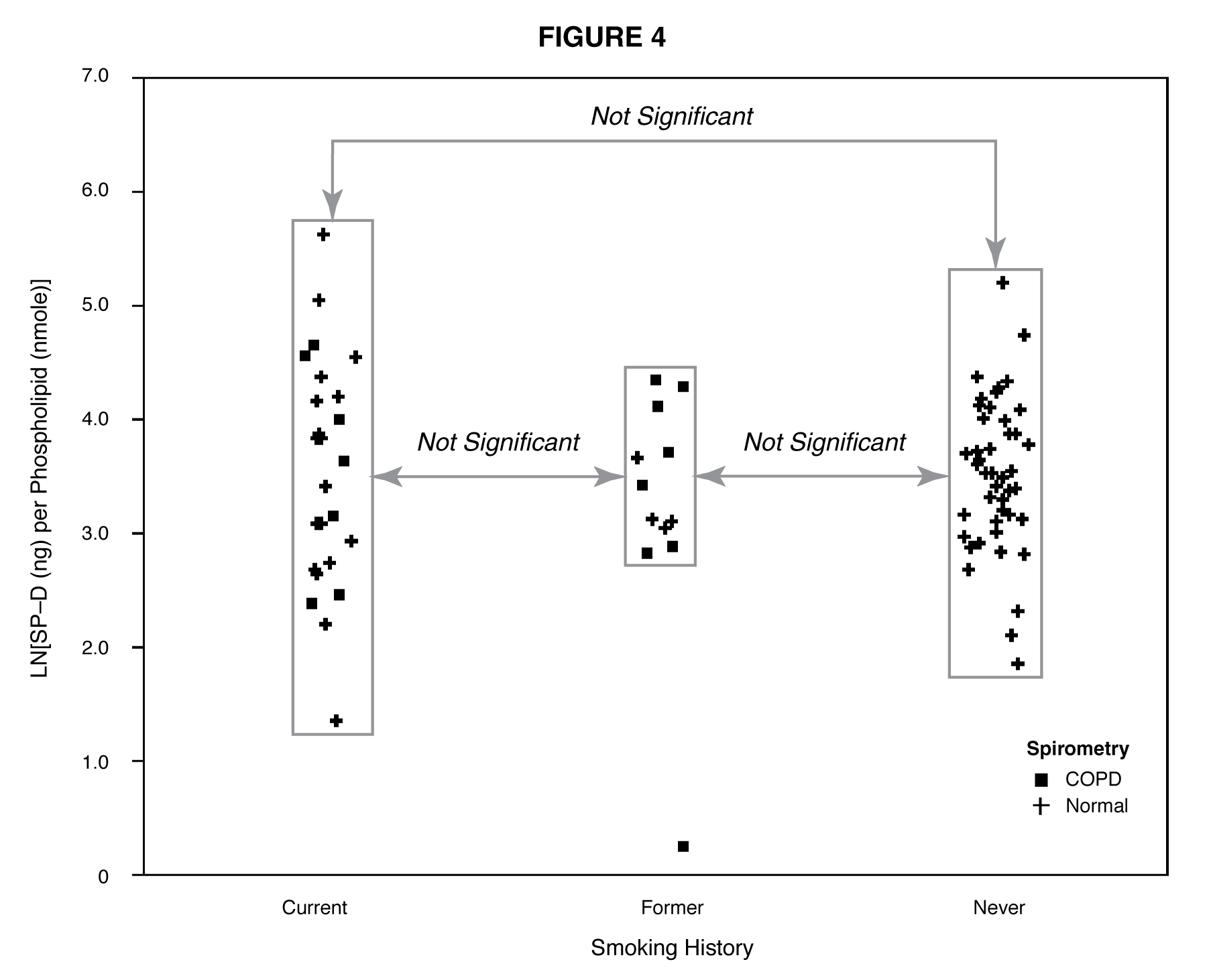

Supplement: Additional file 1 — Natural log transformed data used for analysis. A table summarizing natural log transformed data and four figures showing the distribution of natural log transformed data by group [file 1471-2466-10-53-S1.DOC]
